# Simultaneous Isolation of Lactoferrin and Lactoperoxidase from Bovine Colostrum by SPEC 70 SLS Cation Exchange Resin

**DOI:** 10.3390/ijerph8093764

**Published:** 2011-09-21

**Authors:** Yafei Liang, Xuewan Wang, Mianbin Wu, Wanping Zhu

**Affiliations:** 1 Department of Chemical and Biological Engineering, Zhejiang University, Hangzhou, Zhejiang Province, 310027, China; E-Mails: ihrmyylyf@163.com (Y.L.); wangxwzju@gmail.com (X.W.); 2 People Hospital of Jinhua City, Jinhua, Zhejiang Province, 321000, China; 3 Zhejiang Academy of Traditional Chinese Medicine, Hangzhou, Zhejiang Province, 310007, China; E-Mail: zjuwoo@gmail.com

**Keywords:** defatted colostrum, lactoferrin, lactoperoxidase, SPEC 70 SLS resin, isolation

## Abstract

In this work, simultaneous isolation of lactoferrin (Lf) and lactoperoxidase (Lp) from defatted bovine colostrum by one-step cation exchange chromatography with SPEC 70 SLS ion-exchange resin was investigated. A RP-HPLC method for Lf and Lp determination was developed and optimized as the following conditions: detection wavelength of 220 nm, flow rate of 1 mL/min and acetonitrile concentration from 25% to 75% within 20 min. The adsorption process of Lf on SPEC 70 SLS resin was optimized using Lf standard as substrate. The maximum static binding capacity of SPEC 70 SLS resin was of 22.0 mg/g resin at 15 °C, pH 7.0 and adsorption time 3 h. The Lf adsorption process could be well described by the Langmuir adsorption isotherm model, with a maximum adsorption capacity of 21.73 mg/g resin at 15 °C. In batch fractionation of defatted colostrum, the binding capacities of SPEC 70 SLS resin for adsorbing Lf and Lp simultaneously under the abovementioned conditions were 7.60 and 6.89 mg/g resin, respectively, both of which were superior to those of CM Sepharose F.F. or SP Sepharose F.F. resins under the same conditions. As a result, SPEC 70 SLS resin was considered as a successful candidate for direct and economic purification of Lf and Lp from defatted colostrum.

## 1. Introduction

Lactoferrin (Lf) from bovine colostrum and milk has become increasingly important because of its diverse range of biological activities, such as anti-infective activities toward a broad spectrum of species, antioxidant activities and promotion of iron transfer [1–3]. It also exhibits the potential for chemoprevention of colon and other cancers as a natural ingredient [4]. The expanding market demand for Lf, in particular as an added product based largely on its nutritional values and physiological benefits, has incentivized people to find much more simple and economic ways to separate Lf. The literature regarding the isolation and purification of the Lf during the last two decades is extensive. Reported methods have included microfiltration, gel filtration chromatography, traditional cation exchange chromatography, heparin-agarose affinity chromatography and membrane filtration [5–9]. Despite the increasing number of separation methods that were developed, most of the Lf was obtained from bovine colostrum due to its high content of Lf. Lf separation from regular milk and colostrum using traditional adsorbents was coupled with changes of their physical and chemical properties and flavor, due to the low levels of Lf in regular milk and the high viscosity of colostrums, respectively. The contents of Lf and lactoperoxidase (Lp) in bovine colostrum and regular milk are shown in Table 1. Furthermore, large volumes of adsorption residue must be disposed of as waste products after the proteins of interest are extracted. Such large amounts of disposed proteins caused serious pollution to the environment due to their high BOD (biological oxygen demand) levels [10].

Cation exchange chromatography is the most popular procedure for bovine Lf purification at bLF-supplying companies. However, the packed bed chromatography method, in particular with CM and SP resins, has been identified as having a high pressure drop, and relatively slow intra-bead mass transport due to their smaller particle and pore size distribution [12]. SPEC 70 SLS resin is an anionic chromatography sorbent with a diameter between 260 and 600 μm, which was synthesized by radical copolymerisation of a functionalised monomer (2 acrylamido-2-methylpropanesulfonic acid–AMPS) and a bifunctional crosslinking monomer (*N*,*N*′-methylene-bis-acrylamide–MBA). The active chemical group of SPEC 70 SLS was the sulfopropyl moiety, which was ionized depending upon the pH or ionic strength conditions and adsorbed electrically charged molecules with opposite signs. SPEC 70 SLS resin is very macro-porous and allows a large surface contact and efficient mass transfer. Therefore, the macromolecules diffuse easily from the outside to inside the beads. In addition, good biocompatibility of SPEC 70 SLS avoided its non-specific adsorption and a high separation efficiency of the target protein was obtained. SPEC 70 SLS is approved as a food contact substance by the US FDA (FCN 000325 Notification).

In this work, we described the application of SPEC 70 SLS resin to directly separate Lf and Lp from defatted colostrum. It is shown that Lf and Lp were easily and much more effectively obtained using SPEC 70 SLS resin compared with strong (SP, sulfopropyl) and weak (CM, carboxymethyl) cation exchange resins. In addition, the original properties and flavors of colostrum were preserved. The nontoxicity (referring to FDA FCN000325) of SPEC 70 SLS ensured the milk and colostrum could be used for other applications, for example for the manufacture of other food, after selectively removing of Lf and Lp. Therefore, optimization of the separation conditions of SPEC 70 SLS was of interest in the improvement of colostrum and milk utilization and environmental protection.

## 2. Materials and Methods

### 2.1. Materials

Raw bovine colostrum was obtained from Xiaoshan dairy farm (Hangzhou, Zhejiang Province, China). Skimmed colostrum was prepared from raw colostrum by centrifugation in a Sigma MA3-18 centrifuge at 4,500 r/min for 20 min at 4 °C. After delipidation, skimmed colostrum was used quickly or stored at −20 °C until use. CM Sepharose F.F. and SP Sepharose F.F. were gifts from GE Healthcare (Uppsala, Sweden). SPEC 70 SLS resin were purchased from Pall Corporation (Port Washington, NY, USA).

Bovine Lf, Lp and immunoglobulin G (IgG) standards were purchased from Sigma (St. Louis, MO, USA). Electrophoresis Kits were purchased from Bio-Rad Laboratories, Inc (Benicia, CA, USA). Sodium phosphate buffers and other eluents were degassed after 0.45 μm cellulose acetate membrane filtration. Acetonitrile (MeCN; HPLC grade) and trifluoroacetic acid (TFA; HPLC grade) were purchased from Merck (Darmstadt, Germany). Other reagents were analytical grade or better. Water used for buffer solutions was prepared by water purification system from Stedim arium® 61215 ASTM Type II Pure Water System (Goettingen, Germany).

### 2.2. RP-HPLC

The HPLC system included a LabAlliance Serial III instrument (State College, PA, USA) interfaced with a Model 500 absorbance detector and a JS-3050 data acquisition and manipulation system. Gradient elution was carried out with two solvents. Solvent A was 0.1% (v/v) trifluoroacetic acid (TFA) in DI water and solvent B was 0.1% (v/v) TFA, 95% acetonitrile in DI water, which were extended from the method of Elgar *et al.* [13]. A 1 mL Proteonavi C_4_ column (Shiseido, Tokyo, Japan) was employed with a flow-rate of 1 mL/min at 20. The column was equilibrated with 25% solvent A. After a 10 μL sample was injected, a 1-min isocratic period was followed by a 20-min linear gradient to 75% solvent A. 1-min linear gradient to 25% A was applied after a 1-min hold at 75% A. The column was re-equilibrated with 25% A for 5 min. Detection wavelength was set at 220 nm for the relatively low baseline noise and strong protein signal. A six-point standard curve was constructed from Lf standard solutions at working concentrations of 0.1, 0.2, 0.4, 0.6, 0.8, 1 mg/mL respectively. Prior to RP-HPLC analysis, samples of the colostrum and elution fractions were filtered through 0.45 μm cellulose acetate membrane. The Lf concentration of all samples was adjusted to bring them into the likely range of the standard curve before determination.

### 2.3. Gel Electrophoresis

Desalted elution fractions were analyzed by sodium dodecyl sulphate-polyacrylamide gel electrophoresis (SDS-PAGE). Standards solutions and eluent samples were diluted in sample buffer which contains β-mercaptoethanol to 1 mg/mL and heated in water of 65 °C for 30 min. Load volume of samples was 15 μL. Used the voltage of 80 V at the beginning of electrophoresis, and switched the voltage to 110 V after the sample ran into the separating gel. Protein bands on gels stained with Coomassie Brilliant Blue was imaged and analysed by Quantity One (BioRad Laboratories, Inc (Benicia, CA, USA).

### 2.4. Adsorption and Elution of Lf under the Static Conditions

To investigate the optimal adsorption and elution time of Lf from the bovine colostrum under static conditions, 100 mL pH 7.0 bovine colostrum was incubated in a rotating shaker with 3 g SPEC 70 SLS resin at 4 °C, 230 r/min for 12 h. The time course of adsorption was recorded according to concentrations of Lf in the substrate solutions at different times. After adsorption, the SPEC 70 SLS resin was washed with de-ionized water followed by elution with 300 mL volumes of 330 mmol/L NaCl, 850 mmol/L and 2 mol/L NaCl in series, respectively. The concentrations of Lp and Lf in the 330 mmol/L and 850 mmol/L NaCl eluted solutions, respectively, were analyzed by the RP-HPLC method. The time course of elution was determined according to the concentrations of Lf and Lp at different elution times.

Static binding capacity of SPEC 70 SLS was measured using standard Lf solution as substrate. After equilibration for 3 h with phosphate buffer (pH 7.0), 3 g of each adsorbent was incubated overnight at 4 °C in a rotating shaker (230 r/min) with different concentrations of 100 mL standard Lf solution at, pH 7.0. After adsorption, the SPEC 70 SLS resin was washed with de-ionized water followed by 300 mL 2 mol/L NaCl solution. The binding capacity of each adsorbent was determined by the Lf content in the fraction eluted with 2 M NaCl solution, based on the mass of adsorbent used (dry basis). The Lf concentrations of the elution solutions were assayed using a RP-HPLC method. Binding capacities were determined for different concentrations of pure Lf solutions by the difference between initial and final content of Lf.

The effect of pH and adsorption time on binding capacity was also determined. Sodium phosphate (20 mM) buffers at six pH values (pH 5.0–8.5) were prepared and the static binding capacity of SPEC 70 SLS resin was determined. Three g of pre-equilibrated resin was incubated overnight in 1 mg/mL Lf in binding buffer at each pH. At the initial pH of bovine colostrum, the binding capacity was measured based on the difference between the initial Lf content and that at different times.

### 2.5. SPEC 70 SLS, CM Sepharose F.F. and SP Sepharose F.F. Chromatography Processes

The resin was first equilibrated with 20 mM pH 7.0 sodium phosphate binding buffer. After the equilibrated resin was added into bovine colostrum for 3 h at 15 °C and 230 r/min in a rotary shaker then the wet resin was packed in a column with 6 cm high and an internal diameter of 1.6 cm (Φ 1.6 × 6 cm). The elution process of Lf and Lp adsorbed on cation exchange resins were carried out by a NaCl gradient elution method. The concentration of Lf in each fraction collected was assayed by the RP-HPLC method described above. The same resin was used repeatedly for all chromatography experiments and regenerated with 2 M NaCl solution after each adsorption. The binding capacity was calculated using Equation (1), where Q is the binding capacity, V_L_ was the volume of Lf and Lp fractions collected, C is the concentration of Lf and Lp determined by RP-HPLC and M is dry mass of the SPEC 70 SLS:

(1) $$Q=\frac{C\times V_{L}}{M}$$

## 3. Results and Discussion

### 3.1. Optimization of RP-HPLC Method for Lf and Lp Determination

According to the full-band spectrogram of Lf and Lp, the detection wavelength was set at 220 nm because of the relatively low baseline noise. After that, a Thermo Hypersil BDS C_18_ column and Proteonavi C_4_ column were applied to detect the concentration of Lf in bovine colostrums by a HPLC method. The Proteonavi C_4_ column was selected in this work due to its higher sensitivity and better shape of the elution peak.

**Figure 1:**
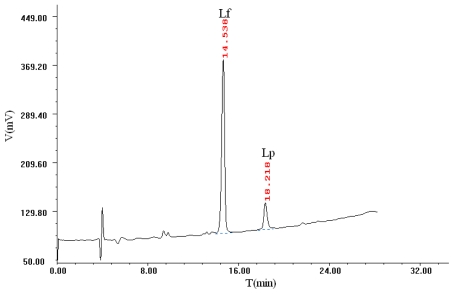
RP-HPLC chomatogram of a Lf and Lp mixture.

Water and acetonitrile were employed as the mobile phase and the gradient and flow rate of the mobile phase were optimized in this study. Due to the high amount of aromatic amino acids (> 9%) in Lf, the absorption of Lf appears relatively high in comparison to that of Lp. In order to easily distinguish the Lf peak from the Lp peak, the concentration of Lf and Lp in the mixture for RP-HPLC were set to 1.0 and 0.5 mg/mL, respectively. The chomatogram is shown in Figure 1. The peaks of fractions were identified by comparison with commercial standards. A linear calibration plot was obtained for the Lf and Lp standards in the concentration range of 0.1–1.0 mg/mL.

### 3.2. Adsorption and Elution Time

**Figure 2:**
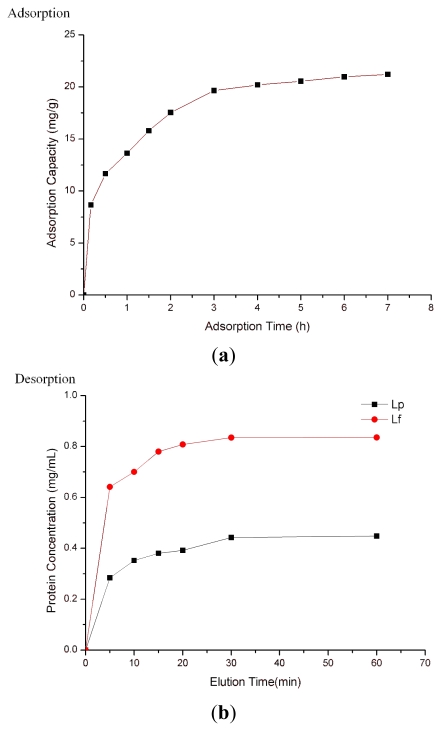
The adsorption (**a**); and desorption (**b**) against time curves of SPEC 70 SLS resin.

Experimental results demonstrated that the binding capacity of SPEC 70 SLS increased from zero to 19.7 mg/g adsorbent after 3 h incubation (Figure 2a). After that, there were no significant differences in the binding capacity, which indicated that 3 h adsorption time was optimal for Lf adsorption under the test conditions. After adsorption, SPEC 70 SLS resin was washed with de-ionized water followed by elution with 330 mmol/L NaCl, 850 mmol/L and 2 mol/L NaCl in series. Figure 2b presents the effects of elution time on desorption of Lf and Lp. As shown in Figure 2b, the optimum elution times for Lf and Lp were 15 min and 30 min, respectively. In view of the ionic strength tolerance, SPEC 70 SLS was regenerated for 5–10 min by 2 M NaCl solution.

### 3.3. Effect of pH on Binding Capacity of SPEC 70 SLS Resin

**Figure 3:**
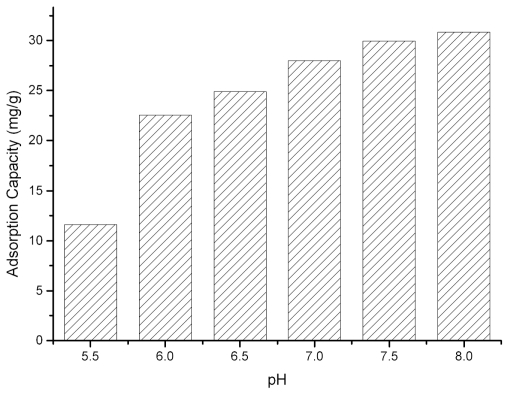
Adsorption of Lf at various pH values on SPEC 70 SLS resin.

Figure 3 shows the binding capacity of SPEC 70 SLS for Lf at different pH values. As the pH increased, higher adsorption capacity values were reached. It was generally expected that the binding capacity would be enhanced as the pH of the buffer deviated downwards from the pI of the Lf, for the Lf would be more highly charged at lower pH values. However, it had been reported [14] that adsorption was most favorable near the pI of the protein, and therefore the maximum adsorption occurred at that pH value. The fact that the net charge concept cannot completely explain the binding properties of proteins to adsorbents alone has been gradually recognized. Surface charge distribution and zeta potential, conformational structure, and the total amount of charged groups in the contact regions will change due to the change of buffer pH. All of these effects will have certain impact on the protein adsorption [12]. Fraaije reported that London-van der Waals force made the protein have a compact structure at the isoelectric point, which favors protein binding to the surface of the adsorbent [15]. In consideration of the pH stability of Lf, the effects of higher pH values (>8) were not measured. In order to preserve original properties and flavors of colostrum, the final adsorption pH was set as pH 7.0.

### 3.4. Binding Capacity of SPEC 70 SLS Resin

**Figure 4:**
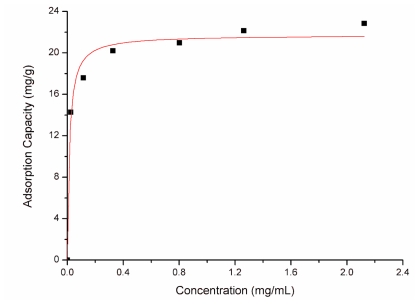
Adsorption isotherms for Lf on SPEC 70 SLS, measured in a standard solution at pH 7.0 and 15 °C.

The equilibrium adsorption isotherm for Lf on SPEC 70 SLS resin is presented in Figure 4. As the initial concentration of Lf increased from zero to 3 mg/mL, the Lf binding capacity went from zero to 22 mg/g adsorbent.

The data were analyzed by Langmuir and Freundlich adsorption isotherm models. The fitted parameters for those two kinds of isotherms are summarized in Table 2. The obtained adsorption isotherm curve of Lf on SPEC 70 SLS at pH 7.0 and 15 °C was of Langmuir type (Figure 4). The maximum Lf adsorption capacity was found to be near the experimental result for SPEC 70 SLS. In addition, Rf had a large value compared with that of Freundlich type. Thus, the measured Lf adsorption isotherm can be reasonably described by a Langmuir isotherm model, with a maximum adsorption capacity of 21.73 mg/g adsorbent at 15 °C.

### 3.5. Isolation of Lf and Lp from Bovine Colostrum

The objective of this work was to use SPEC 70 SLS cation-exchange resin to simultaneously isolate Lf and Lp from regular colostrum efficiently and directly, based on the isoelectric point differences of the proteins (see Table 1). Adsorption experiments were performed at the pH 7.0 of the bovine colostrum in order to insure its physical, chemical and biological properties were not affected. The optimal elution conditions were explored by a linear gradient elution method. A stepwise elution method was then employed to study the elution behavior of the bound proteins under the optimized conditions. The elution profiles and SDS-PAGE image are shown in following figures. The adsorption media showed different selectivity and, consequently, different binding capacity for Lf and Lp in colostrum.

**Figure 5:**
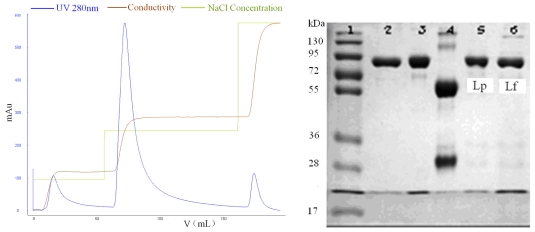
Isolation of LF and Lp in a stepwise manner from the defatted colostrum using SPEC 70 SLS column (Φ 1.6 × 6 cm) after static adsorption and the analysis graph obtained from Quantity One software. SDS-PAGE: Lane 1, standard protein markers; Lane 2, Lf; Lane 3, Lp; Lane 4, IgG; Lanes 5 and 6, the isolates of first and second peaks eluted by different concentrations of NaCl.

**Figure 6:**
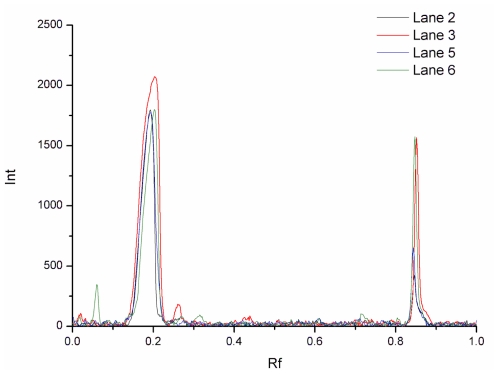
Gel image analysis curves of isolates from SPEC 70 SLS resin.

Figure 5 shows the SPEC 70 SLS chromatography results. Two peaks were obtained after the column was developed with 0–1 M NaCl concentration gradient in buffer. The elution concentrations were 330 mM for the first peak and 850 mM for the second one. The column was then washed with 2 M NaCl and the elution peak, containing a high concentration of salt, was deemed too small for meaningful evaluation. Fractions obtained by a stepwise elution were analyzed with SDS-PAGE (see Figure 5), and further analyzed by a RP-HPLC method. The comparison of lanes 2, 3, 5, 6 is shown in Figure 6. The first peak contained mainly Lp, in addition to much smaller amounts of impurity (possibly immunoglobulin G), and the purity of Lp was 96.6%, according to the optical density analysis using the Quantity One software. The second one contained mainly Lf and the purity of Lf was 91.3%. Lf and Lp were effectively separated due to the considerable difference between their elution concentrations of NaCl.

**Figure 7:**
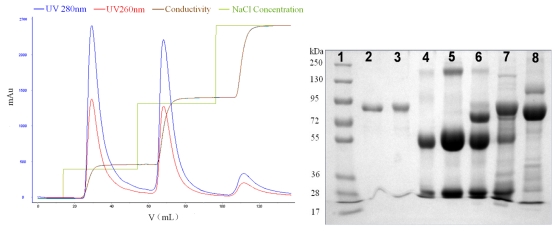
Isolation of Lf and Lp in a stepwise manner from the defatted colostrum using SP Sepharose FF column (Φ 1.6 × 6 cm) after static adsorption. SDS-PAGE: Lane 1, standard protein markers; Lane 2, Lf; Lane 3, Lp; Lane 4, IgG; Lane 5, isolate from whey of colostrum powder; Lanes 6,7 and 8, the isolates of first, second and third peaks.

CM Sepharose F.F. and SP Sepharose F.F. resins showed distinct differences in elution behavior compared with SPEC 70 SLS resin. As known from the gradient elution profile, the optimal NaCl concentrations for the peaks of SP Sepharose FF were 128, 412 and 750 mM, respectively. As can be seen in Figure 7, the 128 mM NaCl fraction (Lane 6) contained mainly IgG.

**Figure 8:**
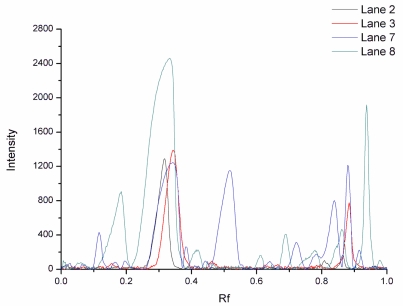
Gel image analysis curves of isolates from SP Sepharose FF resin.

The protein band of Lane 6 with the molecular weight of 75,000 Da was speculated to be IgG consisting of one heavy and one light peptide chains. The IgG purity of the first peak fraction, determined by affinity chromatography, was 86.3%. The comparison of Lanes 2, 3, 7 and 8 using the Quantity One software is shown in Figure 8. With 412 mM NaCl, Lp was eluted and there was a visible IgG band, suggesting that IgG was not thoroughly eluted at 128 mM NaCl. According to results from image acquisition and analysis system, the purity of Lp was of about 67.3%. Therefore, further processing was needed to obtain pure Lp. The fraction in the last peak of SP Sepharose FF was identified as Lf (Lane 8 in SDS-PAGE Figure 7), and the purity was of 73.8%.

**Figure 9:**
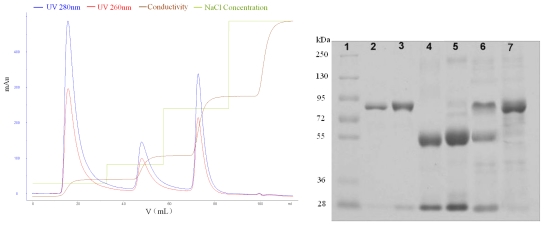
Isolation of Lf and Lp in a stepwise manner from the defatted colostrum using CM Sepharose F.F. column (Φ 1.6 × 6 cm) after static adsorption. SDS-PAGE: Lanes 1, 2, 3, 4 were the same with SP lanes; Lanes 5, 6 and 7, the isolates of the first, second and third peak.

Thee peaks were obtained after a linear gradient elution with the NaCl concentration of 144, 364 and 1,000 mM, respectively. Based on gel image analysis, the main fraction of the first and second CM Sepharose F.F. column effluents was identified as IgG and Lp, respectively. However, IgG and Lp were not completely separated from each other (Figure 9). Besides, the third peak fractions contained a certain amount of impurities, apart from the main Lf component.

The adsorption capability of the adsorbents toward Lf and Lp was investigated and results are shown in Table 3. The binding capacity of SPEC 70 SLS resin for Lf standard was much lower than that of CM and SP. However, the Lf adsorption capacity of SPEC 70 SLS resin was higher than that of CM and SP for bovine colostrum. Comparing the same experimental volume of SPEC 70 SLS, CM and SP resins possessed larger specific surface area due to their smaller bead size. That is, more active chemical groups were crosslinked for the same amount of adsorbent, which was responsible for the distinct difference of adsorption capacity for Lf standard solution. However, the macroporous properties of SPEC 70 SLS allowed for efficient mass transfer and the macromolecules diffused easily from the outside to the inside of the beads. Besides that, SPEC 70 SLS had good selectivity for Lf and Lp. On the contrary, the relative high viscosity of bovine colostrum possibly blocked the pores of the resins due to their smaller pore diameter. Therefore, when it came to bovine whey or colostrum with high viscosity, SPEC 70 SLS possesses special advantages for the separation of Lf and Lp.

## 4. Conclusions

SPEC 70 SLS was designed for the selective separation of proteins with high isoelectric points, avoiding non-specific adsorption. It has broad application prospects due to its biocompatibility and large particle size. Without changing the original physical and chemical properties and flavors of defatted colostrum, the separation of Lf and Lp was achieved by a one step chromatography process.

In the present work, the authors have described the simple preparation method of SPEC 70 SLS which determined that it possesses macroporous properties. The resin provided a higher binding capacity and better selectivity for Lf and Lp in defatted colostrum, compared with CM and SP Sepharose F.F. cation-exchange resins under optimized conditions. Lf and Lp in the effluents were quantitatively detected by RP-HPLC using a Proteonavi C_4_ column during the experiments. The adsorption parameters of Lf standard onto SPEC 70 SLS resin were also investigated. The Langmuir isotherm model described the experimental data well. The direct capture of Lf and Lp from defatted colostrum decreased processing time, avoided the casein removal step and preserved the biological activities of the proteins of interest. Besides, the original properties and flavors of bovine colostrum were preserved after the separation process. The bovine colostrum could be reused for other applications, such as in the food industry, which avoids the environment pollution caused by traditional separation processes.

## Figures and Tables

**Table 1:** Major protein composition in bovine milk and colostrums (Data from [11]).

Parameters	α-La	β-Lg	Lf	Lp	IgG	BSA
pI	4.3–5.1	5.2–5.4	7.8–8.0	9.2–9.9	5.8–7.3	5.13
Mr.	~14,200	~18,400	77,100 ± 1,500	78,000	150,000	66,200
Milk (mg/mL)	0.7–1.8	2.4–4.1	0.02–0.35	11–45	0.3–0.6	~0.3
Colostrum (mg/mL)	1.2–2.4	5.5–19.0	1.5–5	13–30	5–80	0.2–1.2

**Table 2:** Langmuir and Freundlich isotherm model constants of SPEC 70 SLS for Lf adsorption.

Experiment	Langmuir constants			Freundlich constants		
q/(mg/g)	q_m_ (mg/g)	K_d_ (mg/mL)	R^2^	K_F_	n	R^2^
22.0	21.73	0.014	0.9815	21.59	0.099	0.9660

Langmuir model, $q=\frac{q_{m}\times c}{K_{d}+c}$; Freundlich model, *q* = *K_F_* × *c^n^*

**Table 3:** The binding capacity of thee adsorbents for Lf and Lp.

Sample matrix	Adsorption capacity (mg/g)		
	70	SP	CM
Standard (Lf)	24.90	125.49	143.29
Bovine Colostrum (Lf)	7.64	5.81	3.79
Bovine Colostrum (Lp)	6.89	7.48	2.31

## References

[1] Van der Kraan MI, Groenink J, Nazmi K, Veerman EC, Bolscher JG, Nieuw Amerongen AV (2004). Lactoferrampin: A novel antimicrobial peptide in the N1-domain of bovine lactoferrin. Peptides.

[2] Baker EN, Baker HM (2009). A structural framework for understanding the multifunctional character of lactoferrin. Biochimie.

[3] Lönnerdal B (2009). Nutritional roles of lactoferrin. Curr Opin Clin Nutr Metab Care.

[4] Tsuda H, Sekine K, Ushida Y, Kuhara T, Takasuka N, Iigo M, Han BS, Moore MA (2000). Milk and dairy products in cancer prevention: Focus on bovine lactoferrin. Mutat Res.

[5] Brisson G, Britten M, Pouliot Y (2007). Electrically-enhanced crossflow microfiltration for separation of lactoferrin from whey protein mixtures. J Membr Sci.

[6] Zydney AL (1998). Protein separation using membrane filtration: New opportunities for whey purification. Int Dairy J.

[7] Wu MB, Xu YJ (2009). Isolation and purification of lactoferrin and immunoglobulin G from bovine colostrum with serial cation-anion exchange chromatography. Biotechnol Bioprocess Eng.

[8] Ounis WB, Gauthier SF, Turgeon SL, Roufik S, Pouliot Y (2008). Separation of minor protein components from whey protein isolates by heparin affinity chromatography. Int Dairy J.

[9] Almashikhi SA, Nakal S (1987). Isolation of bovine immunoglobulins and lactoferrin from whey proteins by gel filtration techniques. J Dairy Sci.

[10] Liang M, Chen VVYT, Chen HL, Chen WL (2006). A simple and direct isolation of whey components from raw milk by gel filtration chromatography and structural characterization by Fourier transform Raman spectroscopy. Talanta.

[11] Hahn R, Schulz PM, Schaupp C, Jungbauer A (1998). Bovine whey fractionation based on cation-exchange chromatography. J Chomatogr A.

[12] Saufi SM, Fee CJ (2009). Fractionation of β-lactoglobulin from whey by mixed matrix membrane ion exchange chromatography. Biotechnol Bioeng.

[13] Elgar DF, Norris CS, Ayers JS, Pritchard M, Otter DE, Palmano KP (2000). Simultaneous separation and quantitation of the major bovine whey proteins including proteose peptone and caseinomacropeptide by reversed-phase high-performance liquid chromatography on polystyrene-divinybenzene. J Chomatogr A.

[14] Zhao GF, Peng GY, Li FQ, Shi QH, Sun Y (2008). 5-Aminoindole, a new ligand for hydrophobic charge induction chromatography. J Chomatogr A.

[15] Fraaije JG, Norde W, Lyklema J (1991). Interfacial thermodynamics of protein adsorption and ion co-adsorption. III. Electrochemistry of bovine serum albumin adsorption on silver iodide. Biophys Chem.

